# Key Role of Reinforcing Structures in the Flame Retardant Performance of Self-Reinforced Polypropylene Composites

**DOI:** 10.3390/polym8080289

**Published:** 2016-08-08

**Authors:** Katalin Bocz, Dániel Simon, Tamás Bárány, György Marosi

**Affiliations:** 1 Department of Organic Chemistry and Technology, Faculty of Chemical Technology and Biotechnology, Budapest University of Technology and Economics, Műegyetem rkp. 3, Budapest H-1111, Hungary; gmarosi@mail.bme.hu; 2 Department of Polymer Engineering, Faculty of Mechanical Engineering, Budapest University of Technology and Economics, Műegyetem rkp. 3, Budapest H-1111, Hungary; S.Dani012@hotmail.com (D.S.); barany@pt.bme.hu (T.B.); 3 Research Group for Composite Science and Technology, Hungarian Academy of Sciences, Budapest University of Technology and Economics, Műegyetem rkp. 3, Budapest H-1111, Hungary

**Keywords:** self-reinforced polypropylene composite, flame retardancy, intumescence, polymer fibre, mechanical testing

## Abstract

The flame retardant synergism between highly stretched polymer fibres and intumescent flame retardant systems was investigated in self-reinforced polypropylene composites. It was found that the structure of reinforcement, such as degree of molecular orientation, fibre alignment and weave type, has a particular effect on the fire performance of the intumescent system. As little as 7.2 wt % additive content, one third of the amount needed in non-reinforced polypropylene matrix, was sufficient to reach a UL-94 V-0 rating. The best result was found in self-reinforced polypropylene composites reinforced with unidirectional fibres. In addition to the fire retardant performance, the mechanical properties were also evaluated. The maximum was found at optimal consolidation temperature, while the flame retardant additive in the matrix did not influence the mechanical performance up to the investigated 13 wt % concentration.

## 1. Introduction

Self-reinforced composites (SRCs) are a relatively new family of composite materials in which the polymer matrix is reinforced with highly oriented polymer fibres or tapes, both derived from the same polymer type [1]. The basic concept of self-reinforcement is the creation of highly aligned molecular or supramolecular (reinforcing) structures with mechanical properties superior to those of the isotropic (matrix) polymer. As a result of molecular orientation during spinning and drawing, high-performance polymer fibres can be achieved [2] serving as suitable reinforcements in the structurally similar polymer matrix. The high mechanical performance polymer fibres provide some other specific features, such as low density, low cost, recyclability and good interfacial bonding without any surface treatment. The self-reinforced composites, made entirely of highly flammable polymeric components, have found applications in various fields where fire retardancy is a high priority, but until recently no solutions were published for their flame retardation.

Intumescent flame retardants (IFRs) are of general application in polymers, in particularly in polyolefins, and more recently in their fibre-reinforced composites as well. It was proposed by researchers that fibres, depending on their type and chemical structure, may interact with intumescent flame retardant systems. Accordingly, fibres can be classified into three groups: inert or inorganic fibres, organic char forming fibres and non-char forming fibres. Inorganic fibres, such as glass, silica or alumina, are very good protective barriers for flame and heat, as they can withstand temperatures up to 1100 °C for a considerable time. In contrast, conventional organic fibres or textiles are highly flammable due to their high specific surface area, which significantly enhance the rates at which pyrolytic formation of volatile fuels and subsequent combustion occurs. Char-forming organic fibres are either natural, such as cellulose and wool, or synthetic thermoplastics with aromatic structure such as some polyesters and polyamides. Other organic thermoplastic fibres, such as polyolefins, on the other hand, have poor or zero char-forming capability.

When incorporated in a polymer matrix, glass fibres cause a so-called “candlewick effect”, which generally means a big challenge for the flame retardation of the thermoplastic composites [3]. Due to the candlewick effect, glass fibres are able to transfer and feed the fuel from the pyrolysis zone of the polymer matrices to the flame by capillary action, speed the heat flowing back to polymers and thus make the polymers decompose and burn faster. Thus, to achieve a UL-94 V-0 rating, the glass fibre-reinforced thermoplastics need a higher amount of flame retardants than neat polymers do [4]. Liu et al. [5] observed during cone calorimeter tests that long glass fibres destroy the foaming capability of the intumescent flame retardants and the continuity of the residue char, which decreases the flame retardancy of IFR in long-glass-fibre-reinforced polypropylene composites.

Natural fibres, such as ramie and flax, were found to cause a candle-wick effect as well [6,7]. The cellulosic fibres speed the transfer of the flammable mass to the burning area and make the natural fibre reinforced composites more flammable. However, it has been demonstrated by Horrocks [8] that if an intumescent is interspersed within a flame retardant fibrous assembly and both components char, then a so-called “char-bonded structure” may arise. This integrated fibrous-intumescent char structure has a physical integrity superior to those of charred fabric or intumescent alone and, because of reduced oxygen accessibility, demonstrates an unusually high resistance to oxidation when exposed to temperatures above 500 °C. Furthermore, these composite structures show significantly reduced rates of heat release when subjected to heat fluxes of 35 kW/m^2^, thus demonstrating additional significant fire barrier characteristics. Le Bras et al. [9] also showed that association of charring of cellulosic material (flax fibre) and of intumescent system allows for an optimised FR formulation. The effectiveness of the char-bonded structure as a flame and heat barrier is considered to be dependent on the efficiency of the interaction of fibre and intumescent char-forming substances. Similar interactions are not seen with glass fibres and non-char-forming thermoplastic fibres [10].

It is stated by Horrocks [8] that thermoplastic fibres, such as polypropylene (PP) and some polyesters, even when flame retarded using either comonomeric modifications or additives introduced during polymerisation and/or fibre extrusion stages, melt drip and/or form holes when exposed to flame. They cannot, therefore, be used in applications such as protective clothing and barrier textiles, where sustained thermal protection via char formation is an essential requirement. However, our pioneering activity in the field of intumescent flame-retarded self-reinforced composites resulted in some relevant progress. Less than half the amount (9 wt %) of commercial intumescent flame retardant (IFR) additive proved to be sufficient to achieve self-extinguishing behaviour (i.e., V-0 rating) in self-reinforced polypropylene composites compared to the non-reinforced counterparts, owing to a previously unknown flame retardant mechanism [11]. Efficient flame retardancy is provided by a compact surface-protecting layer, which is formed as a result of synergistic coincidence of expansion (foaming), induced by an intumescent flame-retardant, and shrinking (relaxation) of the reinforcing fibres, both initiated by heat of flame. It was proposed that the structure formed this way is influenced by the degree of orientation of the reinforcing polymer fibres [12]. This novel, physical interaction-based flame retardant synergism was utilized in recycled self-reinforced composites, made entirely of secondary polyolefins [13], and also in biodegradable all-poly(lactic acid) composites [14]; however, in the latter case, due to the achievable low draw ratio (λ ~ 4–7) (i.e., low degree of molecular orientation) of poly(lactic acid) fibres, the efficiency was moderate.

In this work, the previously described phenomenon was further investigated in self-reinforced PP composites. The effect of reinforcing structure, i.e., fibre alignment and weave type, and the consolidation quality of multilayer film-stacked [15] composites were examined on the flame retardant performance of intumescent system. Furthermore, to support our earlier theory regarding the key role of molecular orientation of reinforcing fibres, SRCs with identical structure and IFR content but prepared at different consolidation temperatures were investigated, presuming that with increasing processing temperatures the degree of molecular orientation of the highly stretched reinforcing fibres decreases. Considering both the mechanical and flammability performance of the SRCs, we intended to draw conclusions regarding the flame retardant mechanism.

## 2. Materials and Methods 

### 2.1. Materials

A Tipplen R 1059 A (produced by MOL Petrochemicals, Tiszaújváros, Hungary) type random copolymer polypropylene (PP) (*MFI* = 85 g/10 min, 230 °C/2.16 kg, *T*_m_ = 148 °C) was used as the matrix material for the prepared composites.

Two types of PP reinforcing fabrics were used, quasi-unidirectional woven (UD) and plain-woven fabric (PW), each composed of the same highly stretched PP multifilament. The PP multifilament was supplied by Lanex A.S. (Bolatice, Czech Republic), has a melting temperature of *T*_m_ = 170 °C (determined by DSC), a tensile strength of 620 MPa and a tensile modulus of 7800 MPa measured on a single fibre. The areal weight and the mechanical characteristics of the reinforcing structures are summarized in Table 1.

As flame retardant (FR) additive, Exolit AP 766 (produced by Clariant Plastics & Coatings Ltd., Muttenz, Schwitzerland) was added to the matrix material, which is a combined ammonium polyphosphate (APP) and charring agent containing IFR additive. Based on the product datasheet the phosphorus content of the additive is 23.0%–25.0% and its nitrogen content is 14.4%–16.4%.

### 2.2. Preparation of Composites

Reference and flame retardant SRCs with differing reinforcing structures (UD, CP (cross-ply arrangement of UD fabrics) and PW) were prepared with identical layer structures, i.e., 12 reinforcing layers (for CP and PW the layers were arranged in 0/90°) and 13 matrix foils, and with nominal reinforcement contents of 55 wt % by film-stacking method. For this purpose, first reference and flame retarded matrix materials were produced by melt-compounding method with a Labtech Scientific LTE 26-44 co-rotating twin-screw extruder (Labtech Engineering Co. Ltd., Samutprakarn, Thailand; L/D: 44, die temperature: 175 °C, screw rotation speed: 100 1/min). 0, 20 and 30 wt % FR agent was mixed to the neat PP matrix to obtain SRCs with nominal flame retardant contents of 0, 9 and 13.5 wt %, respectively. The extruded materials were cooled down by air cooling method and granulated to 3 mm length (Labtech LZ-120/VS, Labtech Engineering Co. Ltd., Samutprakarn, Thailand). In the second step, from granulates 150 µm thick films were manufactured by Labtech LCR300 film extrusion (Labtech Engineering Co. Ltd., Samutprakarn, Thailand; screw rotation speed: 30 1/min, temperature of the extruder zones: 180, 190, 195 °C, temperature of the coat-hanger die: 195 °C, winding speed: 8 1/min). Then, reference and flame retarded consolidated SRC sheets were manufactured using film-stacking method and compression moulding technique. The film-stacked packages were inserted in between the preheated moulds (170 °C for the first series and 145…190 °C with 5 °C steps for the second series) and held for 120 s without pressure, then compressed for 120 s under a pressure of 4.21 MPa and finally cooled to 40 °C (under pressure) with a cooling speed of 15 °C/min. The thicknesses of the produced SRC sheets ranged between 3.5–4.2 mm. As reference, non-reinforced PP sheets were prepared by straightforward mixing of the required amount of IFR additive (0, 9 and 13 wt %) with PP in a Brabender Plasti Corder PL 2000 type (Brabender GmbH., Duisburg, Germany) internal mixer at 190 °C with a rotor speed of 30 rpm for 10 min. The blends were then hot pressed to form 4 mm thick plates in a Collin P200E type (Dr Collin GmbH, Munich, Germany) laboratory hot press.

### 2.3. Characterization Methods

#### 2.3.1. Density Measurements

The density of the specimens (ρ_s_) of 13 × 25 mm^2^ dimension was obtained from weight measurements in air and water and calculated according to Equation (1), where *m*_a_ is the specimen’s weight in air, *m*_e_ is the specimen’s weight in ethanol and ρ_e_ is the density of ethanol at room temperature equal to 0.789 g/cm^3^.

(1) $$\rho_{s}=\;\frac{m_{a}}{m_{a}-m_{e}}\rho_{\text{e}}\;\left[\frac{g}{{\text{cm}}^{3}}\right]$$

#### 2.3.2. Mechanical Characterisation

Peel tests were performed on rectangular specimens of 25.4 mm × 250 mm using Zwick Z250 (Zwick GmbH & Co. KG, Ulm, Germany) universal testing machine according to the ASTM D 3167-97 standard with a crosshead speed of 150 mm/min. 

Comparative static tensile tests were performed on rectangular specimens of 25 mm × 200 mm. The tensile tests were carried out by a universal ZWICK Z250 testing machine (Zwick GmbH & Co. KG, Ulm, Germany). The cross-head speed was set to 5 mm/min.

All the above listed mechanical tests were performed at room temperature and at least five specimens were tested in all cases. All specimens were cut by water jet.

#### 2.3.3. Characterisation of the Fire Behaviour

Standard UL-94 flammability tests (according to ASTM D3081 and ASTM D635, respectively) were carried out in order to classify the samples based on their flammability in horizontal and vertical test setups. The sample size was 120 mm × 13 mm.

Mass loss type cone calorimeter tests were carried out by an instrument made by FTT Inc. using the ISO 13927 standard method. Specimens (100 mm × 100 mm) were exposed to a constant heat flux of 50 kW/m^2^ and ignited. Heat release values and mass reduction were continuously recorded during burning. In all cases 3 samples of identical compositions were tested.

The fire behaviour of the reference and flame retarded systems was characterized by limiting oxygen index measurements (LOI, according to ASTM D2863). The LOI value expresses the lowest volume fraction of oxygen in a mixture of oxygen and nitrogen that supports flaming combustion of a material under specified test conditions. The sample size was 120 mm × 13 mm.

## 3. Results and Discussion

### 3.1. Investigation of the Effect of the Self-Reinforcing Structure on the Mechanical and Flammability Properties

Three types of self-reinforced composites composed of different PP reinforcing structures, i.e., UD (unidirectional), CP (cross-ply) and PW (plain woven), were prepared with identical 55 wt % nominal reinforcement content and under identical manufacturing conditions (170 °C) at three flame retardant levels, at 0, 9 and 13 wt % nominal additive contents, respectively. It has to be noted that the actual reinforcement and additive content of the prepared composites, due to the differing areal weight of the reinforcements and some loss of matrix material during hot-pressing, slightly differs from the set nominal values. Therefore, the actual mass fractions of reinforcement and flame retardants were determined by mass measurements in all cases.

**Figure 1:**
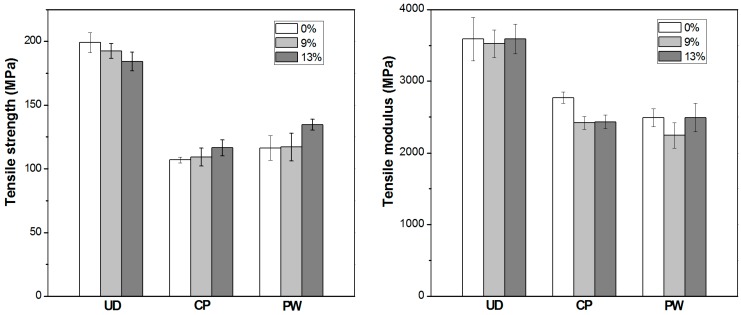
Tensile strength and modulus of additive-free and flame retarded PP SRCs with UD, CP and PW reinforcement.

The mechanical performance, expressed by tensile strength and tensile modulus values, are shown in Figure 1. As expected, the tensile strength and modulus values are related to the ratio of fibres aligned in the load bearing direction. It can also be seen that the flame retardant content of the matrix layers does not significantly influence the mechanical performance of the composites, which is in full agreement with our previous results [11,13]. Based on the tensile test results, adequate consolidation quality of the self-reinforced composites can be assumed independently of reinforcing structure and additive content.

**Figure 2:**
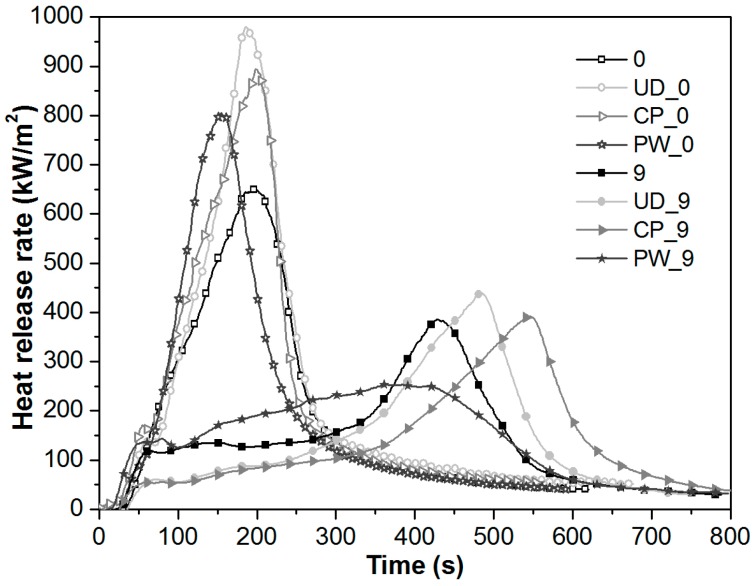
Heat release rate curves of non-reinforced and self-reinforced PP samples with flame retardant contents of 0 and 9 wt %.

The burning behaviour of self-reinforced composites with different reinforcing structures was characterized by mass loss-type cone calorimeter tests. In this test, the heat flux is applied perpendicular to the layers of highly stretched reinforcing fibres. In Figure 2, for better visibility, only the results of the additive-free and 9 wt % flame retardant containing samples are shown. Nevertheless, as a function of reinforcing structure, similar trends were found for the 13 wt % additive containing samples as well. It can be seen, that in additive-free form the burning of self-reinforced composites is accompanied with higher peaks of heat release rate (pkHRR) than the non-reinforced PP reference (0), which is attributed to the inherently higher heat release rate (HRR) of the reinforcing isotactic PP fibres (55–60 wt % of the SRCs) than that of the random copolymer matrix material. Among the SRCs, the highest pkHRR was recorded for the UD reinforced composite, while the lowest for the PW one. Moreover, the different reinforcing structures influenced the burning behaviour of the composites differently. In the case of UD and CP alignment of reinforcing fibres, the time of peak heat release rate (pkHRR_time_) was not remarkably influenced, but due to the plain-woven structure (PW) the pkHRR appeared 42 s earlier than in the case of the non-reinforced PP. It is noteworthy that the dissimilar burning characteristics of the chemically identical SRCs are only explainable by physical factors.

Similar trends were observed for the 9 wt % (and 13 wt %) FR containing samples. It can be seen in Figure 2 that the shape of HRR curves of UD_9 and CP_9 are similar to that of non-reinforced 9, however, in the first 300 s of burning significantly lower heat release rates were recorded for the multilayered composites. The pkHRR of the UD_9 and CP_9 composites is slightly higher and shifted in time. In contrast, no sharp peak of heat release rate can be observed for the PW_9 composite, which indicates dissimilar burning behaviour caused by different foaming processes.

In the case of the non-reinforced PP (9) and the UD (UD_9) and CP (CP_9) reinforcing structures, a fairly thick but loose heat insulating foam forms in the initial stage of burning that protects the underlying polymer from combustion. The heat protective efficiency of this foam can be preserved until the degradation of the protective shield begins. This is indicated by the sharp increase in heat release rate as a significant amount of PP gets into the burning zone abruptly. In contrast to this, it was found that the PP plain-woven fabrics, similarly to inorganic fabrics [5,16] hinder the development of a highly expanded foam structure. When this multilayer composite is exposed to heat, the shrinkage of the highly stretched, interweaving fibres occurs simultaneously with the intumescence of the flame retarded matrix layers, and thus a more compact charred layer forms on the surface of the PW_9 sample. As the heat insulating property of an intumescent char mainly depends on its thickness (thicker layer gives more insulation), the formation of compact foams in the case of PW_9 is associated with higher initial heat release rates. However, as compact char layers maintain their heat protective character longer than thick but weak chars, the pkHRR is much lower for compact chars. The significantly reduced pkHRRs in the case of multilayer plain-woven fabric reinforced SRCs can be explained by the reduced oxygen permeability, enhanced heat barrier capability and increased strength of the compact char layer formed, which is similar to the “char bonded” structure [17,18] reported in case of fibre–intumescent interaction earlier. It seems that the shrinkage of the interweaving, highly stretched fibres promotes the formation of a coherent network of expanding and interlinked domains by increasing the specific concentration within the surrounding matrix, as predicted by the models proposed by Zhang [19] and Bourbigot [20]. The effectiveness of the observed synergism obviously depends on the ratio of the expanding domains (i.e., FR content) and the shrinking ability (i.e., degree of molecular orientation) which needs to be optimized in order to fully utilize this interaction.

Standard UL-94 tests were performed to compare the ignitability and combustion behaviour of the non-reinforced PP and the three types of self-reinforced composites at three flame retardant levels. The obtained UL-94 ratings are summarized in Table 2 together with the actually measured mass fractions of reinforcement contents of the prepared composite plates, and thus the calculated actual flame retardant contents. During UL-94 test, the heat was applied parallel with the direction of the weft fibres of the SRCs, which actually take 52 wt % in the case of the UD, while 26 wt % in the case of the CP and the PW fabric reinforced composites, respectively (see Table 1).

It can be seen, that in the case of the non-reinforced PP samples 9 and 13 wt % FR content proved to be sufficient only to reach HB and V-2 ratings, respectively, which are in perfect agreement with the literature based expectations [19]. In contrast, each self-reinforced PP composites proved to be self-extinguishing at 13 wt % nominal FR content both in horizontal and vertical position reaching V-0 classification. Furthermore, the SRC with unidirectionally aligned fibres (UD_9) reached V-0 rating even at surprisingly low, at 7.2 wt % additive content, which is about one third of the amount of IFR additives (about 21 wt %) that would be needed in a non-reinforced PP matrix [11]. The results shown in Table 2 suggest that increasing the number of fibres which are aligned in the direction of heat source provides better flame retardant performance to the self-reinforced polymer composites. (Presumably higher reinforcement content would result in even better fire characteristics.)

It was proposed earlier [12] that the degree of molecular orientation of aligned fibres plays a key role in the flame retardant synergism between highly stretched PP fibres and intumescent flame retardant system. In order to further investigate this phenomenon, UD reinforced composites with 9 wt % nominal (and approximately 7.2 wt % actual) IFR content were prepared at different consolidation temperatures ranging between 145 and 190 °C. It is presumed that with increasing processing temperatures, the degree of molecular orientation of highly stretched reinforcing fibres decreases. The morphological, mechanical and flammability properties of this series are investigated hereinafter.

### 3.2. Investigation of the Effect of Molecular Orientation of Aligned Fibres on the Flame Retardant Performance of Self-Reinforced Composites

**Figure 3:**
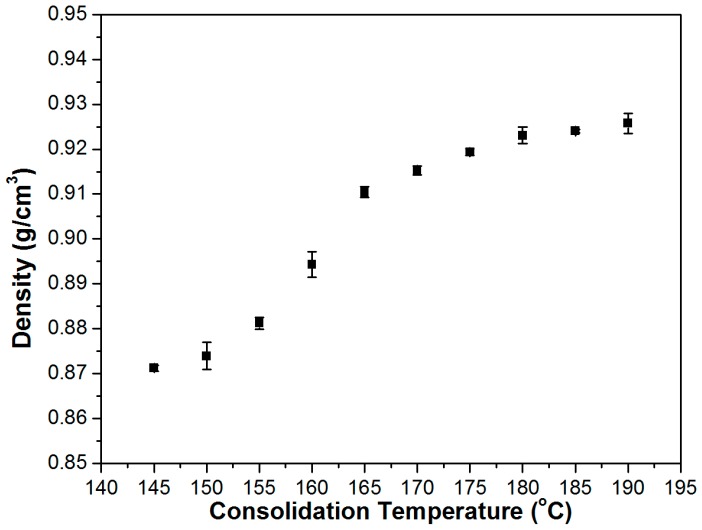
Density of the UD SRCs prepared at different consolidation temperatures.

Density measurements were carried out to characterize the consolidation quality of UD SRCs manufactured between 145 and 190 °C. As it can be seen in Figure 3, the actual density values of the prepared SRCs increased monotonically with increasing consolidation temperature, which indicates less void fraction and better consolidation quality as a function increasing processing temperature.

**Figure 4:**
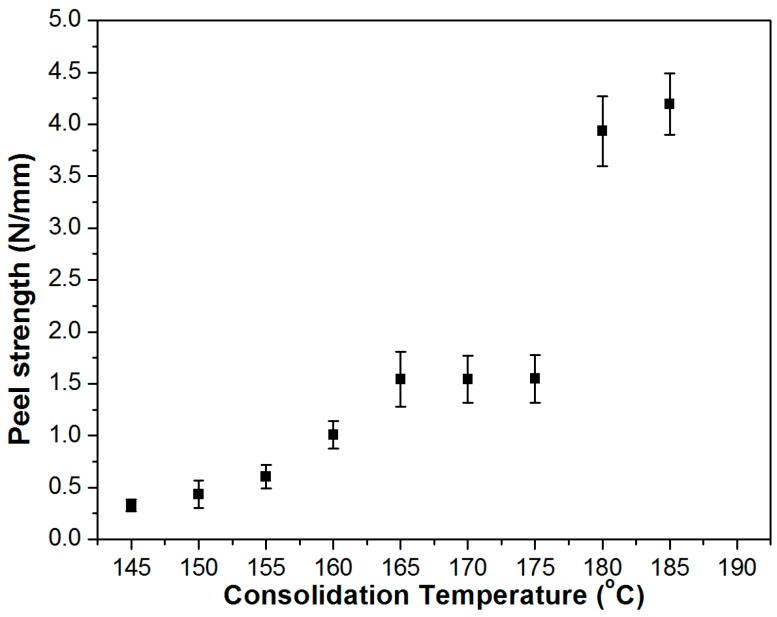
Peel strength of the UD SRCs prepared at different consolidation temperatures.

The interlayer bonding was quantified by peel tests. In Figure 4, the peel strength of composites is plotted against their consolidation temperatures. Accordingly, the peel strength reaches a plateau of 1.5 N/mm around the melting temperature of the reinforcing fibre (*T*_m_ = 170 °C), when the adhesive bonding between fibre and matrix significantly increases providing enhanced interfacial bonding. At a consolidation temperature of 180 °C or higher, the fusion of fibre-matrix interface becomes so significant that the peel strength increases above 4 N/mm.

**Figure 5:**
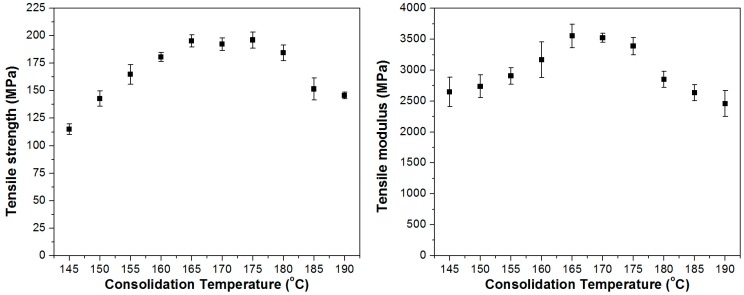
Tensile strength and modulus of the UD SRCs prepared at different consolidation temperatures.

The tensile strength and modulus values are influenced both by the consolidation quality, i.e., fibre-matrix interaction, and by the strength and modulus of the reinforcing PP fibres. The latter ones are mainly determined by the inherent draw ratio (degree of molecular orientation) and the thermally induced relaxation occurred during composite preparation. As a result of all these effects, bell-shaped curves were obtained when the tensile strength and modulus values were plotted against the consolidation temperature of the flame retarded UD SRCs (Figure 5). From 145 to 170 °C the mechanical properties of the SRCs increased with the increasing fibre-matrix interaction and improving consolidation quality. Around 170 °C a tensile strength of about 200 MPa and a tensile modulus of 3500 MPa was reached. At higher temperatures, the increasing mobility of polymer chains, trying to return to the thermodynamically stable coil state, results in noticeable relaxation of molecular orientation and thus a significant loss in mechanical performance.

The effect of molecular orientation of reinforcing fibres on the burning characteristics of intumescent flame retarded UD composites was investigated as well. Regarding the heat release rate curves of the composites, no remarkable change was found; the pkHRR values varied between 395 and 445 kW/m^2^, while the pkHRR_time_ ranged between 415 and 495 s without any tendency as a function of consolidation temperature. Such variation of these fire parameters can be considered to be within the deviation of the characterization method. It was concluded that under the conditions of a cone heater the structure (i.e., woven or non-woven fibres) and layering of reinforcing substances have particular effect on the foaming process and burning behaviour of intumescent flame retarded SRCs, but the effect of consolidation quality of the composites and molecular orientation state of the reinforcing fibres is negligible.

**Figure 6:**
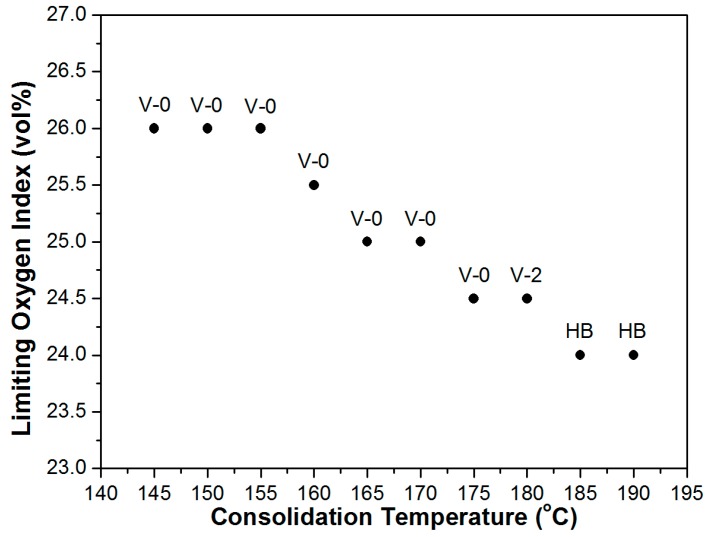
Limiting oxygen index and UL-94 rating of the UD SRCs prepared at different consolidation temperatures.

LOI measurements and standard UL-94 tests were carried out to characterize the ignitability and flammability behaviour of the flame retarded UD composites prepared at different processing temperatures. The obtained results are presented in Figure 6. As can be seen, both the LOI values and the UL-94 ratings show decreasing tendency as a function of consolidation temperature. The highest LOI value of 26 vol% was reached for the composites prepared at the lowest temperatures, between 145 and 155 °C, suggesting that the density and consolidation quality do not affect the flame retarding synergism between the highly stretched PP fibres and the intumescent flame retardant system. It has to be highlighted that a LOI value of 26 vol% was reached in the case of UD reinforced SRCs with flame retardant content of only 7.2 wt %, while in the case of the 9.0 wt % additive containing simple PP mixture a LOI of 24 vol% was measured. Nevertheless, based on our earlier investigation, the difference between the measured LOI values of flame retarded SRCs and PP mixtures would significantly increase with increasing additive loading [11]. As is shown in Figure 6, above the consolidation temperature of 155 °C the LOI values of the flame retarded UD SRCs started to decrease, quasi linearly with the processing temperature. However, the composites showed self-extinguishing behaviour and were classified according to the UL-94 standard as V-0 even up to the consolidation temperature of 175 °C. At higher processing temperatures, despite the identical layer structure and chemical composition, the LOI values decreased to 24 vol % and the UL-94 classification remained HB, which were actually expected values based on the chemical composition (92.8 wt % PP and 7.2 wt % IFR) of the samples.

According to our earlier suggestion [12], it is proposed that the relaxation ability, i.e., the degree of molecular orientation, plays a key role in the prominent flame retarding efficiency of SRCs consisting of highly stretched polymer fibres. With increasing consolidation temperatures the molecular relaxation of the reinforcing fibres during processing increasingly dominates the performance. Consequently, in the case of the SRCs prepared above 175 °C, the remaining relaxation ability becomes insufficient to form a compact charred surface, which would be essential to provide enhanced flame retardant efficiency. Nevertheless, it is presumed that at lower processing temperatures (below 170 °C), where the fibres are still in a highly oriented state, even lower additive content would suffice to reach a V-0 rating.

In principle the LOI values should correlate with the tensile modulus of the composites, as both parameters are remarkably affected by the degree of molecular orientation of the reinforcing fibres. However, the tensile moduli of the SRCs proved to be influenced by other, hardly separable, factors such as density, interfacial interaction and consolidation quality as well. Special methods need to be found in order to characterize the orientation state of the reinforcing fibres of SRCs separately. In the case of adequate correlation, the estimation (or manipulation) of flammability properties would be feasible as well. For this purpose, the authors propose the application of polarized Raman spectroscopy, a fairly sensitive and non-destructive method to study regularity and structure of iPP fibres [21,22,23].

## 4. Conclusions 

In this work, the mechanism behind the prominent flame retardant behaviour of self-reinforced PP composites with fairly low additive contents was investigated.

Three types of PP reinforcing structures, quasi-unidirectional fabrics, cross-ply alignment of quasi-unidirectional fabrics and plain-woven fabrics were layered between flame-retardant additive containing matrix layers with the aim of investigating the effect of reinforcing structures on the flame retardant performance of self-reinforced composites. At a consolidation temperature of 170 °C, well-consolidated, high strength composites were obtained independently from their reinforcing structure and flame retardant content.

It was found that under the cone heater, where the heat is applied perpendicular to the layers of highly stretched reinforcing fibres, the structure of reinforcement has a particular effect on the foam forming process and burning behaviour of intumescent flame-retarded, self-reinforced composites. On the one hand, the combustion of UD and CP reinforced SRCs is accompanied by lower initial heat release rates and in time shifted peaks of heat release rate compared to those of the non-reinforced PP counterparts with identical flame retardant contents. On the other hand, very dissimilar heat release rate curves, with higher initial values but significantly (by 20%) reduced peaks of heat release rate, are characteristic for the PW fabric reinforced flame retardant SRCs. It is proposed that plain-woven fabrics, similarly to inorganic fabrics, hinder the formation of a thick but loose heat insulating foam, but contribute to the formation of a compact charred layer of increased thermal stability and mechanical strength providing improved fire protection.

The effect of reinforcing structures proved to be different under UL-94 and LOI circumstances, where the heat was applied parallel with the direction of the weft fibres of the SRCs. A V-0 rating was reached at 13 wt % nominal flame retardant content for all three types of SRCs; however, for the UD reinforced composite, as little as 7.2 wt % additive sufficed to ensure self-extinguishing behaviour (V-0 rating), which is about one third of the amount of IFR additives (about 21 wt %) that is normally necessitated in non-reinforced PP matrix. These results confirm our earlier assumption that the degree of molecular orientation of reinforcing fibres (here the number of fibres aligned in the heat direction) plays a key role in the flame retardant performance.

The results of UD reinforced composites prepared at different consolidation temperatures ranging between 145 and 190 °C also confirmed this phenomenon. With increasing processing temperatures the degree of molecular orientation of highly stretched reinforcing fibres decreased. Density measurements and peel tests provided evidence for improving consolidation quality of flame retarded UD SRCs with increasing processing temperature. The tensile strength and modulus values showed a bell-shaped tendency as a function of consolidation temperature; up to 170 °C the mechanical performance increased mainly due to the increasing fibre-matrix interaction, while at higher temperatures the heat induced relaxation of molecular orientation caused noticeable deterioration of the mechanical properties.

Based on the cone calorimeter test results, it was concluded that the effect of consolidation quality of the composites and the molecular orientation state of reinforcing fibres is negligible regarding the heat release of the flame retarded SRCs. On the other hand, the relaxation ability, i.e., the degree of molecular orientation, of the reinforcing PP fibres proved to be crucial regarding the flammability characteristics during UL-94 tests and LOI measurements. The lower the consolidation temperature applied, the better the flame retardant performance that was achieved. A 7.2 wt % flame retardant containing UD SRCs reached a V-0 rating up to the processing temperature of 175°C. At higher consolidation temperatures, the remaining relaxation ability of fibres became insufficient to compensate for the intumescence of the flame retardant additive and thus to form a special compact charred surface, which would be essential to provide enhanced flame retardant efficiency.

It is proposed that the novel synergy demonstrated between highly oriented polymer fibres and an intumescent flame retardant system could be utilized in other polymer types/systems to provide a more cost-effective fire retardancy solution.

## Figures and Tables

**Table 1:** Areal weight and mechanical characteristics of the reinforcing structures.

Type of fabric	Linear density of the components (dtex) (ratio)		Areal weight (g/m^2^)	Tensile strength (MPa)	
	Warp	Weft		Warp	Weft
**UD fabric**	550 (14%)	2,200 (86%)	181		264 ± 6
**PW fabric**	550 (53%)	550 (47%)	178	249 ± 2	222 ± 3

**Table 2:** UL-94 ratings of additive-free and flame retarded non-reinforced PP and PP SRCs.

Composite	Reinforcement content (wt %)	Flame retardant content (wt %)	UL-94 rating
**0**	0.0	0.0	HB
**9**	0.0	9.0	HB
**13**	0.0	13.0	V-2
**UD_0**	58.2	0.0	HB
**UD_9**	60.9	7.2	V-0
**UD_13**	60.7	10.8	V-0
**CP_0**	58.4	0.0	HB
**CP_9**	60.9	7.2	HB
**CP_13**	60.9	10.8	V-0
**PW_0**	53.0	0.0	HB
**PW_9**	55.5	8.9	HB
**PW_13**	55.1	13.5	V-0

## References

[1] Karger-Kocsis J., Bárány T. (2014). Single-polymer composites (SPCs): Status and future trends. Compos. Sci. Technol..

[2] Dabrowska I., Fambri L., Pegoretti A., Slouf M., Vackova T., Kolarik J. (2015). Spinning, drawing and physical properties of polypropylene nanocomposite fibers with fumed nanosilica. Express Polym. Lett..

[3] Chen Y.H., Wang Q. (2006). Preparation, properties and characterizations of halogen-free nitrogene-phosphorous flame-retarded glass-fiber reinforced polyamide 6 composite. Polym. Degrad. Stabil..

[4] Zhao C.S., Huang F.L., Xiong W.C., Wang Y.Z. (2008). A novel halogen-free flame retardant for glass-fiber-reinforced poly(ethylene terephthalate). Polym. Degrad. Stabil..

[5] Liu Y., Deng C.L., Zhao J., Wang J.S., Chen L., Wang Y.Z. (2011). An efficiently halogen-free flame-retardant long-glass-fiber-reinforced polypropylene system. Polym. Degrad. Stabil..

[6] Li S., Ren J., Hua Y., Yu T., Weizhong Y. (2010). Influence of ammonium polyphosphate on the flame retardancy and mechanical properties of ramie fiber-reinforced poly(lactic acid) biocomposites. Polym. Int..

[7] Szolnoki B., Bocz K., Sóti P.L., Bodzay B., Zimonyi E., Toldy A., Morlin B., Bujnowicz K., Wladyka-Przybylak M., Marosi G. (2015). Development of natural fibre reinforced flame retarded epoxy resin composites. Polym. Degrad. Stabil..

[8] Horrocks A.R. (1996). Developments in flame retardants for heat and fire resistant textiles-the role of char formation and intumescence. Polym. Degrad. Stabil..

[9] Le Bras M., Duquesne S., Fois M., Grisel M., Poutch F. (2005). Intumescent polypropylene/flax blends: A preliminary study. Polym. Degrad. Stabil..

[10] Horrocks A.R., Anand S.C., Sanderson D. (1996). Complex char formation in flame retarded fibre-intumescent combinations: 1. Scanning electron microscopic studies. Polymer.

[11] Bocz K., Bárány T., Toldy A., Bodzay B., Csontos I., Madi K., Marosi G. (2013). Self-extinguishing polypropylene with a mass fraction of 9% intumescent additive–A new physical way for enhancing the fire retardant efficiency. Polym. Degrad. Stabil..

[12] Bocz K., Igricz T., Domonkos M., Bárány T., Marosi G. (2013). Self-extinguishing polypropylene with a mass fraction of 9% intumescent additive II-Influence of highly oriented fibres. Polym. Degrad. Stabil..

[13] Bocz K., Toldy A., Kmetty Á., Bárány T., Igricz T., Marosi G. (2012). Development of flame retarded self-reinforced composite from automotive shredder plastic waste. Polym. Degrad. Stabil..

[14] Bocz K., Domonkos M., Igricz T., Kmetty Á., Bárány T., Marosi G. (2015). Flame retarded self-reinforced poly(lactic acid) composites of outstanding impact resistance. Compos. Part A.

[15] Halász I.Z., Romhány G., Kmetty Á., Bárány T., Czigány T. (2015). Failure of compression molded all-polyolefin composites studied by acoustic emission. Express Polym. Lett..

[16] Toldy A., Szolnoki B., Marosi G. (2011). Flame retardancy of fibre-reinforced epoxy resin composites for aerospace applications. Polym. Degrad. Stabil..

[17] Kandola B.K., Horrocks A.R. (1996). Complex char formation in flame-retarded fibre intumescent combinations-II. Thermal analytical studies. Polym. Degrad. Stabil..

[18] Kandola B.K., Horrocks S., Horrocks A.R. (1997). Evidence of interaction in flame retardant fibre-intumescent combinations by thermal analytical techniques. Thermochim. Acta.

[19] Zhang S., Horrocks A.R. (2003). A review of flame retardant polypropylene fibres. Prog. Polym. Sci..

[20] Bourbigot S., Le Bras M., Delobel R. (1993). Carbonization mechanisms resulting from intumescence association with the ammonium polyphosphate-pentaerythritol fire retardant system. Carbon.

[21] Fraser G., Hendra P., Watson D., Gall M., Willis H., Cudby M. (1995). The Raman spectra of oriented isotactic polypropylene. Spectrochim. Acta Part A.

[22] Ran S., Fang D., Sics I., Toki S., Hsiao B.S., Chu B. (2003). Combined techniques of Raman spectroscopy and synchrotron X-ray for in-situ studies of polypropylene fibers during tensile deformation. Rev. Sci. Instrum..

[23] Kida T., Hiejima Y., Nitta K.H. (2016). Molecular orientation behavior of isotactic polypropylene under uniaxial stretching by rheo-Raman spectroscopy. Express Polym. Lett..

